# Sugarcane Biorefinery from Component Separation to High-Value Outputs: Technical Progress and Future Perspectives

**DOI:** 10.3390/foods15111877

**Published:** 2026-05-26

**Authors:** Jiaxuan Dai, Jing Chen, Bo Lin, Liyu Lu, Fengjin Zheng, Krishan K. Verma, Ganlin Chen

**Affiliations:** 1 College of Light Industry and Food Engineering, Guangxi University, Nanning 530004, China; m19912616163@163.com; 2 Guangxi Subtropical Crops Research Institute, Guangxi Academy of Agricultural Sciences, Nanning 530001, China; jchen1030@gxaas.net (J.C.); 2417393015@st.gxu.edu.cn (L.L.); 3 Key Laboratory of Quality and Safety Control for Subtropical Fruit and Vegetable, Ministry of Agriculture and Rural Affair, Nanning 530001, China; 4 Guangxi Key Laboratory of Quality and Safety Control for Subtropical Fruits, Nanning 530001, China; 5 Institute of Agro-Products Processing Science and Technology, Guangxi Academy of Agricultural Sciences, Nanning 530007, China; linbo@gxaas.net; 6 College of Agriculture, Guangxi University, Nanning 530004, China; 7 Sugarcane Research Institute, Guangxi Academy of Agricultural Sciences, Nanning 530007, China; drvermakishan@gmail.com

**Keywords:** sugarcane processing, bioproducts, component utilization, sugarcane juice and bagasse, filter mud

## Abstract

As a major global sugar crop and lignocellulosic feedstock, sugarcane processing traditionally suffers from single-product dependency and low byproduct utilization, causing resource waste and environmental factors. To address this, the ‘sugarcane processing tree’ framework offers a pathway for full-component valorization. This review systematically summarizes the high-value utilization pathways for sugarcane juice, bagasse, and filter mud. Key quantitative insights reveal that the functional sugars offer high profitability due to premium market prices; bagasse pretreatment constitutes 40–50% of overall biorefinery costs; and crude wax recovery from filter mud stagnates at only 5–8%, limiting commercial scale-up. Current bottlenecks are characterized by low pretreatment efficiency, subpar strain performance, and high isolation costs. Future advancements must integrate coupled biorefining, synthetic biology, and standardized frameworks to spearhead the low-carbon, circular transition of the sugarcane industry for sustainable development.

## 1. Introduction

**Figure 1:**
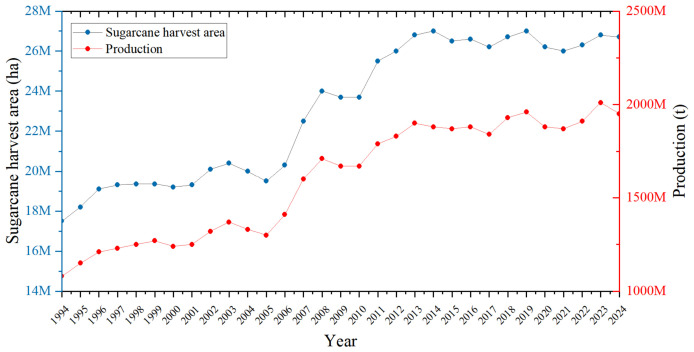
The area of sugarcane harvest and production globally, based on reference [1].

**Figure 2:**
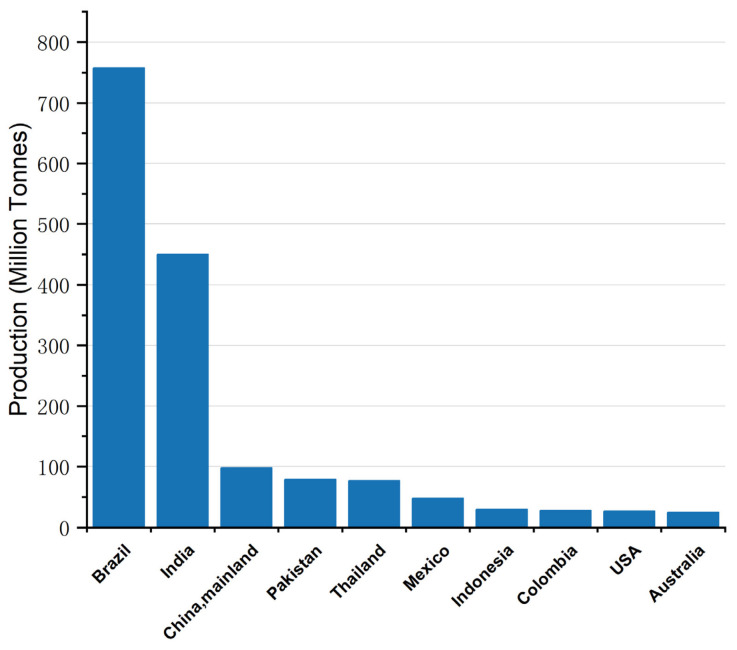
Top 10 sugarcane-producing countries, with Brazil, India, and China leading production of 2024 [1].

Sugarcane (*Saccharum* spp. Hybrids) is the global primary sugar crop and a significant lignocellulosic biomass resource. Approximately 86% of the world’s sugar is produce from sugarcane, forming a vast cultivation and processing industrial system across over 100 tropical and subtropical countries, including Brazil, India, and China. According to Food and Agriculture Organization of the United Nations (FAOUN), global sugarcane production and harvested area have shown pronounced long-term growth over the past 30 years (1994–2024), with total yield surging by more than 80% from around 1.1 billion tons to nearly 2.0 billion tons (Figure 1). In 2024, global sugarcane output surpassed 1.8 billion tons. Regionally, the Americas supplied 51.4% (predominantly driven by Brazil’s production of ~759 million tons), followed by Asia at 42.1%, where China served as a key driver with an output of ~102 million tons. Africa and Oceania held marginal shares of 4.9 and 1.6%, respectively [1] (Figure 2). China is the world’s third-largest sugar producer. Sugarcane sugar production accounts for over 90% of the total domestic sugar output, with the Guangxi province production area contributing nearly 70% [2]. The sugarcane industry has become a pillar of ensuring national sugar security and supporting rural revitalization and green agricultural development in southern China.

A core model characterizes traditional sugarcane processing focused on sugar production and low-value utilization of byproducts [3]. This process generates ca. 279 mts of byproducts annually, including bagasse, molasses, and filter mud. Bagasse, which accounts for 24–27% of the total sugarcane mass, is used for about 60% of its volume, utilized merely for combustion to generate electricity [4]. Another 20% is used for papermaking, making it difficult to achieve the graded, high-value conversion of its three major components, i.e., cellulose, hemicellulose, and lignin [5]. Furthermore, about 30 mts of filter mud are mostly returned directly to the fields, which not only carries risks of pathogen transmission and heavy metal accumulation but also results in the waste of high-value-added components, such as crude wax [6,7]. This industrial model, defined by single-product sugar production and low resource utilization, restricts economic growth and exerts significant environmental pressure [8,9,10,11,12], falling well short of the requirements for low-carbon transformation and circular economy development under carbon-neutrality goals.

In this context, the concept of ‘Sugarcane Processing Tree’ has emerged. It regards sugarcane as a biomass system that can be split into multiple stages for stepwise value addition. Through crushing and separation, three major high-value branches are formed, such as sugarcane juice, bagasse, and filter mud, which extend into diversified product chains including sugar products, functional sugars, bio-based materials, bioenergy, and agricultural materials. The ultimate goal is to achieve ‘full-component utilization, zero waste, and maximum value extraction’. With the growing demand for functional foods, the accelerated substitution of petrochemical products with bio-based materials, and the rapid iteration of biorefining technologies, sugarcane processing is upgrading from single sugar production toward a multi-generation, high-value, and low-carbon direction.

Currently, extensive research has been conducted nationally and internationally on the high-value utilization of sugarcane juice, bagasse, and filter mud across various fields, including food, chemicals, materials, energy, and agriculture. However, current research remains scattered and fragmented, with existing reviews predominantly focusing on single products or byproducts rather than providing a systematic overview from the perspectives of the entire industrial chain, all components, and entire life cycle. This systematic review evaluates research progress in sugarcane biorefinery over the period of 2015–2025, focusing on the high-value utilization of sugarcane juice, bagasse, and filter mud. By integrating literature data and comparing technical routes, this study identifies key bottleneck technologies and outlines future perspectives for the sustainable development of cane agro-industries.

## 2. Overview of Sugarcane Processing Tree

**Figure 3:**
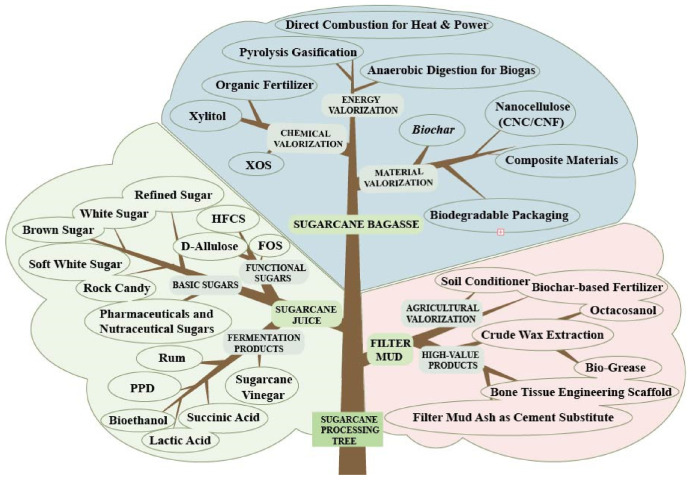
Layout of sugarcane processing.

Globally, current sugarcane research is highly fragmented, predominantly focusing on isolated technical pathways or single-stream waste treatments. To bridge this knowledge gap and provide a unified macro-perspective, the ‘sugarcane processing tree’ framework systematically synthesizes these scattered global studies into a single, cohesive structural map (Figure 3). Mechanically, the sugarcane stalk undergoes mechanical deconstruction via crushing to partition it into sucrose-rich juice and recalcitrant fibrous bagasse matrix, while residual filter mud is isolated during juice clarification. Distinct from conventional biorefineries that inefficiently incinerates bagasse and discard filter mud, this model redefines these streams as parallel valorization pathways. Each intermediate fraction serves as a strategic developmental node branching into specialized downstream chains, ultimately constructing a multi-tiered product portfolio spanning functional foods, bioenergy, biomaterials, sustainable agriculture, and pharmaceuticals. By consolidating isolated technological islands into an interconnected network, Figure 3 functions as a comprehensive visual tool for decoding the sugarcane bio-economy.

### 2.1. Raw Material Composition and Separation

Fresh sugarcane stalks have a moisture content of about 70–75%. The sugarcane juice obtained by crushing is rich in soluble sugars, primarily sucrose, glucose, and fructose, which serve as the core constituents. In addition, sugarcane juice contains various nutrients, including vitamins, proteins, amino acids, and organic acids, as well as trace elements, such as iron (Fe) and zinc (Zn). Furthermore, it is abundant in functional compounds with high nutritional value, including flavonoids, polyphenols, and octacosanol, and exhibits both nutritional and functional properties [13].

Accounting for 24–27% of the total cane mass, sugarcane bagasse is a typical lignocellulosic feedstock characterized by significant regional and seasonal compositional variations. Geographical factors, such as soil chemistry, agronomic management, and climatic variables drive the variability in its primary polymers (cellulose, hemicellulose, and lignin) [14]. On a temporal scale, the continuous lignification of cell walls during the harvesting season accelerates the accumulation of recalcitrant crystalline cellulose and hydrophobic lignin at the expense of hemicellulose. Because these fractions inherently function as a structural composite within the plant matrix, they impart excellent baseline mechanical properties to downstream biopolymers. Compared with energy-restricted traditional incineration, transforming bagasse into biomaterials represents a superior closed-loop biorefinery paradigm dedicated to total-biomass valorization.

**Figure 4:**
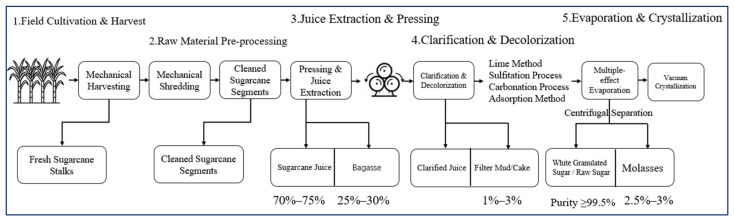
Schematic representation of production process for sugarcane byproducts.

Filter mud is the solid byproduct generated during the sugarcane juice clarification process, primarily consisting of precipitates formed during lime or sulfurous acid clarification. Its composition varies depending on the clarification process, with major components including organic matter (40–60%), crude wax (6.70–11.01%), nitrogen (1–2%), phosphorus (1–3%), potassium (0.5–1.5%), calcium (5–10%), and trace elements, such as iron and magnesium [15]. Filter mud also contains residual sucrose, proteins, colloidal substances, and microbial metabolites. The homologous origin and close process integration of these three components (juice, bagasse, and filter mud) make them highly suitable for coupled co-production and integrated biorefining (Figure 4).

### 2.2. Primary Utilization Pathways

The utilization of sugarcane juice is based on traditional sugar production and extends to refined sugar, functional sugars, sugar alcohols, fermented beverages, bioethanol, and organic acids. It represents the branch with the optimum added values and the most advance industrialization. Since sugarcane juice is rich in fermentable sugars and may be utilized directly without complex pretreatment, it has become a preferred raw material for biological fermentation and food processing.

The utilization of bagasse has expanded beyond traditional applications, such as combustion for power generation, animal feed, and papermaking, to include high-value products, i.e., nanocellulose, activated carbon, biochar, xylose, xylitol, lactic acid, and bioethanol. This represents the most active branch of current research. Due to it’s a variety of output, low cost, and high cellulose content, bagasse is an unique feedstock for second-generation (2-G) biorefining; however, the recalcitrance of its lignocellulosic structure remains the primary obstacle to its efficient utilization.

The utilization of filter mud is based on soil improvement and organic fertilizers, expanding into biochar, fulvic acid fertilizers, crude wax, bio-lubricants, and bone tissue engineering scaffolds. It represents the branch with significant synergistic benefits for sustainable agriculture and environment. Filter mud is rich in organic matter and minerals, with both agricultural and industrial value. However, the management and control of its safety risks remain a prerequisite for its widespread application. Overall, the sugarcane processing tree achieves a transformation ‘from low value to high value, from single products to multi-product co-production, and from end-of-pipe treatment to source-oriented valorization’. It represents the core model for the high-quality development of the sugarcane industry in the future.

## 3. Deep Processing Products

### 3.1. Diversified and High-Value Utilization of Sugarcane Juice

**Figure 5:**
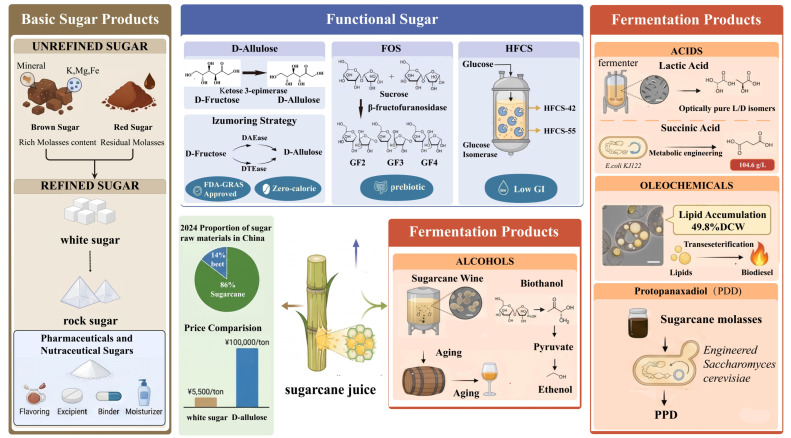
Diversified utilization of sugarcane juice.

Sugarcane juice is rich in soluble sugars, with a simple composition that is easily utilized by microorganisms, making it a high-quality raw material for food processing and biological fermentation. Its utilization pathways can be classified into three major categories, such as basic sugar products, functional sugars, and fermented products (Figure 5). Significant differences exist in the process routes and technical maturity of these products are summarized in Table 1.

#### 3.1.1. Processing of Basic Sugar Products

White granulated sugar is produced from sugarcane juice or raw sugar through processes, such as clarification, concentration, crystallization, centrifugal separation, and drying. It has the advantages of pure white appearance; uniform crystal morphology and high chemical purity, ranking first in both output and application scope among domestic sugar varieties. During the 2024/25 sugar production season, the output of superior and first-grade white granulated sugar accounted for 91.49% of the total national sugar production. It is primarily utilized in beverage production, food processing, catering, seasoning, and daily household use, and also serves as a core asset in future markets and bulk commodity trading [16].

Refined sugar is purified through multiple processes to remove impurities and non-sugar components, resulting in high purity and light color [17]. With the control of trace impurities and harmful components, it is suitable for high-end food, infant formula, and medical preparations that require high quality, accounting for about 2.38% of total production. It has even higher purity, with a sucrose content of ≥99.8%. Characterized by a regular crystalline structure, it is categorized into monocrystalline and polycrystalline types. Due to its clean, sweet taste, it is commonly used in braising, seasoning, traditional tonics, soups, stews, beverages, and as a medicinal ingredient [18]. Brown slab sugar and cube sugar are specialty processed sugar products suitable for niche scenarios, such as Cantonese desserts and coffee or tea service, respectively. Soft white sugar is made by adding invert sugar syrup to fine white granulated sugar. It features small grains, a soft texture, and high solubility with a mild sweetness, making it more suitable for direct consumption [19]. It is common in household consumption and pastry-making in Northern China, accounting for nearly 3.27% of production during the 2024–2025 crushing season.

Non-centrifugal sugars, including raw sugar, brown sugar, and dark brown sugar (black sugar), are produced by extracting juice from raw materials, such as sugarcane or sugar beet, followed by preliminary clarification, evaporation, and crystallization, without undergoing deep decolorization or refining. Different from refined sugar, this type of product retains natural minerals, vitamins and polyphenols from sugarcane, endowing it rich layered flavor and certain nutritional activity. Raw sugar is a brown, viscous product formed after the purification, concentration, and crystallization of sugarcane juice, typically containing sucrose (97.52%), moisture (0.48%), and non-sucrose (2%) impurities [20]. It serves as the intermediate raw material for sugar refining. Brown granulated sugar (Red granulated sugar) is produced from sugarcane or raw sugar liquor after centrifugal separation, retaining some molasses components. It presents a reddish-brown or yellowish-brown color, with a robust flavor, and is frequently used for coloring in baking, seasoning dishes, and making traditional desserts, accounting for 2.57% of production. Brown and dark brown sugar are produced by direct concentration and crystallization of sugarcane juice; their mineral content, such as potassium, magnesium, iron, and calcium, is significantly higher than refined white sugar [33]. Research indicates that the total phenolic content in brown sugar can reach 200–400 mg GAE/100 g, exhibiting antioxidant activity and leading to its wide application in traditional foods and tonics [21,22].

Pharmaceutical sucrose, such as pharmaceuticals and nutraceutical sugars is a critical pharmaceutical excipient, serving as an essential flavoring agent, filler (diluent), binder, and humectant in drug formulations. Pharmaceutical-grade sucrose appears as colorless crystals or a white crystalline loose powder with a sweet taste. As a high-frequency excipient in the medical field, its annual consumption in pharmaceutical preparations reaches as high as 500,000 to 600,000 tons [23]. Furthermore, as an amorphous sugar matrix, it is frequently utilized in the pharmaceutical industry as excipient, bulking agent, and stabilizer for unstable components, such as proteins [24].

#### 3.1.2. Deep Processing of Functional Sugars

Although basic sugar processing technologies are mature and demonstrate significant economies of scale, they face challenges, such as low added value, severe product homogenization, and narrowing profit margins. In China, the average ex-factory price of white sugar in 2023 was nearly 5500 RMB/ton. In contrast, the market price for functional sugars like D-allulose can exceed 100,000 RMB/ton, highlighting a stark difference in added value. With the advancement of the ‘Healthy China’ strategy and the growing popularity of low-sugar diets, traditional high-calorie sucrose is facing substitution pressure. Consequently, functional sugars represented by D-allulose, fructo-oligosaccharides (FOS), and high-fructose corn syrup are rising rapidly, becoming the core direction for the deep processing of sugarcane juice [34].

Among these, D-allulose, a C-3 epimer of D-fructose, serves as a prominent sucrose substitute. It possesses 70% of the sweetness of sucrose but with extremely low caloric content (only 0.4 kcal/g). Characterized by its zero-calorie profile, non-glycemic nature, and non-cariogenic properties, it is currently one of the most promising sugar substitutes [25,26]. Due to its low natural abundance, which cannot meet commercial demands, biosynthetic technology has become the primary pathway for its acquisition. Currently, the core strategy for industrial production involves using inexpensive D-fructose as a substrate, catalyzed by D-psicose 3-epimerase or D-tagatose 3-epimerase. This transformation occurs under mild reaction conditions and yields high product purity, making it suitable for large-scale production [27]. This process, known as the ‘Izumoring’ strategy, has become a research hotspot in the field. The US FDA approved D-allulose as a GRAS (Generally Recognized as Safe) substance in 2012, and it is widely utilized in beverages and baked goods in Japan and South Korea.

Fructo-oligosaccharides (FOS) are fructose oligomers composed of fructose units linked by β-2,1-glycosidic bonds, typically with a degree of polymerization (DP) of 3–5. As a classic prebiotic, FOS can promote the proliferation of beneficial intestinal bacteria and improve gut health [28]. Its role as a prebiotic enhances the intestinal environment and stimulates the growth of Bifidobacteria, providing significant benefits to human health. The enzymatic synthesis of FOS relies primarily on fructosyltransferase (FTase) or β-fructofuranosidase [29]. However, wild-type enzymes often face challenges, i.e., low catalytic activity and susceptibility to product inhibition by glucose [30]. Through computer-aided rational design, enzyme activity can be significantly enhanced. Chen et al. [30,35] successfully increased the FOS conversion rate of a mutant enzyme from 0.17% in the wild type to 52% through a two-step modification process. Subsequently, further research was conducted to address the glucose inhibition issue identified during the modification process. By constructing a dual-enzyme co-catalytic system involving glucose isomerase and a mutant fructosyltransferase, glucose inhibition was mitigated, further increasing FOS synthesis by 55.51% [30].

Due to their physiological functions and processing characteristics, fructo-oligosaccharides (FOS) are widely used in food, healthcare products, animal feed, and medical applications [36]. In the food industry, it is frequently added to infant formulas, dairy products, baked goods, and sugar-free beverages, where it serves as a sucrose substitute to reduce calories and improve flavor and texture [37]. In the healthcare and pharmaceutical sectors, FOS selectively promotes the proliferation of beneficial bacteria, such as *Bifidobacterium* and *Lactobacillus*, while inhibiting the growth of pathogens, thereby improving intestinal microecology [38]. In the feed industry, it serves as a green, antibiotic-free additive that supports intestinal health, enhances growth performance, and reduces aquaculture pollution, aligning with requirements for enviro-friendly farming [39]. FOS becomes a functional oligosaccharide with broad application prospects for deep processing and high-value utilization of sugarcane in major producing regions, such as Guangxi, China. It will effectively drive the upgrading of the sugar industry from simple sugar production toward multi-functional food ingredients.

High-fructose corn syrup (HFCS), primarily composed of glucose and fructose, is extensively utilized across the beverage, baking, and dairy industries due to its premium sweetness, exceptional solubility, and refreshing flavor profile [31]. Conventionally, HFCS production is starch-centric, manufactured through four core processing stages, such as liquefaction, saccharification, isomerization, and refining [32]. Crucially, owing to its abundance of sucrose, sugarcane juice represents not only a highly advantageous alternative feedstock for HFCS synthesis but also significantly streamlines the production workflow. This transition successfully accomplishes a high-efficiency valorization pathway that repurposes sugar milling streams into premium functional sweeteners.

Overall, functional sugars possess high added value and rapid market growth, serving as a critical pathway for the transformation of sugarcane juice from ‘bulk raw material’ to ‘high-end food ingredient’. It is predicted that the global functional sweetener market will grow from $20 billion in 2023 to over $30 billion by 2030, with a compound annual growth rate (CAGR) of about 6%.

#### 3.1.3. Biological Fermentation Products

Sugarcane juice can be used directly as a fermentation carbon source without the need for complex pretreatment. It is highly suitable for the production of high-value-added fermented products, such as alcoholic beverages, vinegar, bioethanol, and organic acids. To visually present the production characteristics and application differences of sugarcane juice fermented products, Table 2 compares the strains, yields and process parameters of different fermentation products.

##### Sugarcane Wine

Sugarcane wine is a low-alcohol beverage produced by fermenting sugarcane juice and is represented globally by products, such as Cachaça, Basi, and Rum [48,49]. The production process primarily involves juice extraction, sterilization, and alcoholic fermentation. While *Saccharomyces cerevisiae* remains the primary driver of fermentation, recent research has focused on isolating superior indigenous strains with enhanced tolerance to environmental stress and optimal metabolic profiles for volatile compounds [52,53,54]. Other yeast species, such as *Wickerhamomyces anomalus*, also contribute to the flavor complexity through glycerol metabolism. However, the process is susceptible to contamination by wild yeasts and lactic acid bacteria (LAB), which compete for nutrients and reduce ethanol yields. To mitigate this, natural antimicrobials like oregano and thyme essential oils have been explored. These oils exhibit synergistic effects that inhibit LAB while increasing alcohol production by about 3% [50]. Furthermore, co-cultivation of *S. cerevisiae* and *Lactococcus lactis* has shown that bacterial presence, under controlled conditions, can occasionally enhance sensorial characteristics favored by consumers [55].

##### Sugarcane Rum

Sugarcane rum, commonly referred to as ‘Cachaça’ in Brazil, is a distilled spirit produced by fermenting, distilling, and aging sugarcane juice [56,57]. In recent years, extensive scientific research has emerged its production optimization, quality control, flavor chemistry, and sustainability. During alcoholic fermentation, the selection of yeast strains influences the final flavor profile. Co-fermentation optimizes the volatile profile over standard monoculture. Floral-fruity esters shoot up 3-fold. Monoterpene alcohols rise by 50%. Pivotal alcohols increase by 4.5-fold. Conversely, acetic acid plummets by 60%. This shift delivers a richer, less acidic flavor with enhanced sensory attributes [58]. The maturation and traditional oak barrel aging are slow processes [59]. Research has explored accelerated aging approaches using ultrasound-assisted extraction combined with white oak chips. The optimized process enables rapid extraction of polyphenols, achieving color and total phenolic index levels comparable to traditionally aged brands for 12–18 months, thereby significantly shortening the maturation cycle [60].

##### Sugarcane Vinegar

Sugarcane vinegar is an acidic condiment produced from sugarcane juice or molasses through a two-stage fermentation process, i.e., alcoholic fermentation followed by acetic acid fermentation [61,62,63]. During the initial stage, *Saccharomyces cerevisiae* converts fermentable sugars into ethanol. Studies have demonstrated that ethanol yield is positively correlated with sugar concentration; optimized conditions typically involve adjusting sugar levels (12–18%) at pH 4.5 and 28 °C to maximize efficiency [64,65]. In the subsequent acetic fermentation stage, *Acetobacter aceti* oxidizes ethanol to acetic acid, a critical step in determining the final acidity and flavor profile [62]. Furthermore, cell immobilization, such as hosting Acetobacter on wood chip matrices boosts acetic acid productivity by around 75% relative to traditional free-cell methods [61]. This bio-transformation process not only yields a unique flavor but also preserves the functional nutrients inherent in sugarcane.

##### Lactic Acid

Sugarcane juice is an ideal substrate for lactic acid (LA) production due to its high content of fermentable sugars (sucrose, glucose, and fructose), which require no complex pretreatment [66,67,68]. As a critical platform for polylactic acid (PLA) synthesis, LA is primarily produced via lactic acid bacteria (LAB) or engineered microorganisms. *Lactobacillus plantarum* has shown excellent adaptability to sugarcane juice, where medium optimization can significantly enhance biomass and yield production [69]. Furthermore, a novel isolate, *Pediococcus pentosaceus* HLV1, enables highly efficient bioconversion of sugarcane bagasse residues to lactic acid, delivering a remarkable >30% increase in production efficiency [43]. Metabolic engineering and adaptive evolution are essential strategies for improving LA titer and optical purity. By knocking out byproduct pathways and redirecting metabolic flux, LA accumulation can be maximized. For instance, the engineered *Saccharomyces cerevisiae* TAM strain increased L-lactic acid production from 29.8 to 50.5 g/L by downregulating the ethanol and glycerol pathways while enhancing carboxylate transporter expression [44]. Furthermore, adaptive evolution strategies have facilitated the development of thermotolerant strains capable of producing L-lactic acid with high optical purity [70].

##### Succinic Acid

Succinic acid is a high-value chemical with extensive applications in polymers, pharmaceuticals, and food sectors [71]. Rich in fermentable sugars, sugarcane juice serves as a cost-effective substrate for the bio-based production of succinic acid. Since many conventional strains cannot directly utilize sucrose, metabolic engineering has become a primary research focus. By introducing non-phosphotransferase system (non-PTS) sucrose utilization genes into *E. coli* strains (e.g., *AFP111* and *KJ122*), researchers have successfully conferred sucrose-metabolizing capabilities. These engineered strains achieved succinic acid yields of 56–62 g/L using sugarcane molasses [45,46]. To mitigate the high downstream processing costs associated with neutral-pH fermentation, attention has shifted to acid-tolerant yeast strains. Metabolic engineering of *Issatchenkia orientalis*, involving the deletion of byproduct pathways and transporter engineering, enabled efficient production at low pH. This strain achieved a concentration of 104.6 g/L in fed-batch fermentation using sugarcane juice and maintained yield of 63.1 g/L during 300-fold pilot-scale scale-up. This low-pH mode eliminates the need for alkali neutralization, simplifying downstream recovery [47]. Additionally, engineered *Klebsiella oxytoca*, refined through rational design and adaptive evolution, demonstrated robust industrial potential by producing 0.87 g/g of succinic acid directly from untreated sugarcane molasses without supplemental nutrients [72].

##### Ethanol

Sugarcane juice, a renewable resource rich in fermentable sugars, such as sucrose, glucose, and fructose, serves as an ideal substrate for bioethanol production [40,73]. The core of current research and industrial application lies in the fermentation process, where microorganisms-primarily yeasts, specifically *Saccharomyces cerevisiae*-convert these sugars into ethanol [41]. *S. cerevisiae* is the dominant selection for sugarcane juice fermentation due to its efficient sugar metabolism, high ethanol tolerance, and well-established industrial track record. Beyond traditional strains, *Meyerozyma caribbica* MJTm3 has demonstrated significant tolerance to various stress conditions; following the optimization of fermentation parameters via Response Surface Methodology (RSM), it can achieve high ethanol yields near theoretical limits [42].

Genetic engineering of industrial *S. cerevisiae* strains has become a key driver in enhancing ethanol production. For instance, modifying sucrose metabolism specifically by eliminating extracellular invertase activity while overexpressing intracellular invertase and enhancing transport allows strains to consume disaccharides directly without releasing monosaccharides into the medium. This strategy has resulted in 11% increase in ethanol yield compared to parental strains [40]. Furthermore, overexpression of specific genes, such as MSN2, has been shown to improve ethanol tolerance and productivity in industrial yeast strains under high sugar concentrations [74].

##### Bio-Oil

The production of microbial lipids (Single cell oil, SCO) from sugarcane juice is a prominent focus in biorefining. This pathway has demonstrated superior environmental benefits in biodiesel production; pilot-scale tests indicate that SCO-based biodiesel significantly reduces emissions of CO_2_, CO, and NO_x_ compared to soybean-based alternatives, while maintaining competitive production costs [75]. Beyond biofuels, SCOs rich in polyunsaturated fatty acids (PUFAs) and carotenoids are increasingly utilized in high-value sectors, such as nutraceuticals, cosmetics, and the synthesis of oleogels for the confectionery industry [76,77]. Research has identified various oleaginous microorganisms, including yeasts, microalgae, and filamentous fungi, capable of utilizing sugarcane substrates. Notably, *Rhodosporidium toruloides* achieved a lipid content of 49.8% in 1000 L bioreactors using sugarcane juice [75]. While *Saccharomyces cerevisiae* and *Pichia guilliermondii* can utilize molasses, the former yields substantially more lipids [78]. However, heterotrophic cultivation of *Chlorella* sp. and the scale-up of filamentous fungi like *Mucor plumbeus* to 1000 L scales further highlight the industrial feasibility of converting sugarcane-derived carbon into high-value lipids [79,80].

##### Protopanaxadiol

Protopanaxadiol (PPD) is a common precursor to numerous ginsenosides and possesses significant medicinal value. Traditional extraction methods from *Panax* species are inefficient and unsustainable. A recent research breakthrough involving metabolically engineered *Saccharomyces cerevisiae* that can efficiently synthesize PPD using sugarcane molasses as an inexpensive carbon source. The engineered strain, BY-V, achieved a yield of 1.55 ± 0.02 g/L in shake flasks and reached 15.88 ± 0.65 g/L in a 5-liter bioreactor, demonstrating immense potential for industrial-scale production [51].

#### 3.1.4. Challenges

Currently, research on the high-value utilization of sugarcane juice predominantly centers on enhancing the yields of target products, such as functional sugars and organic acids. However, critical bottlenecks persist during industrial fermentation, microbial strains generally exhibit poor tolerance to high sugar, ethanol inhibition, and environmental fluctuations. However, the production costs of key enzymes required for functional sugar synthesis remain prohibitively high. Furthermore, the risk of wild microbial contamination and the seasonal variations in sugarcane juice quality further impede stable, large-scale industrial applications.

### 3.2. High-Value Utilization Pathways for Sugarcane Bagasse

**Figure 6:**
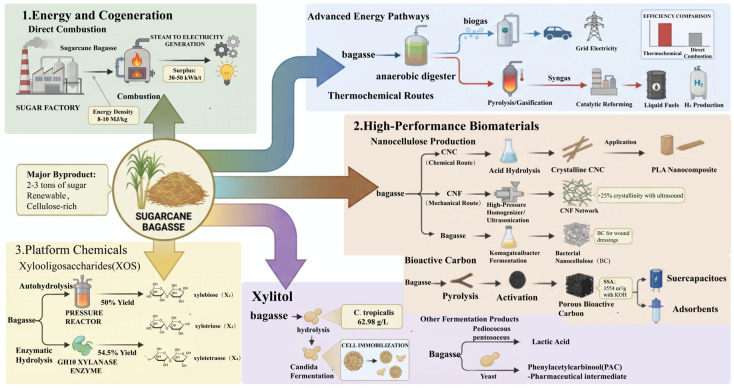
Diversified utilization of sugarcane bagasse.

Sugarcane bagasse is the primary byproduct of the sugar industry, with approximately 2–3 tons generated for every ton of sucrose produced [81]. Rich in cellulose, hemicellulose, and lignin, it represents a low-cost, renewable, and abundant lignocellulosic resource. Traditionally, bagasse has been used mainly for boiler combustion to generate heat and electricity, with low efficiency and minimal added value. In recent years, the utilization of bagasse has rapidly evolved toward three major sectors, such as bio-energy, materials, and chemicals, becoming a core growth driver for the comprehensive, high-value utilization of all sugarcane resources (Figure 6).

#### 3.2.1. Energy, Combined Heat and Power Utilization

Sugarcane bagasse has an energy density of 8–10 MJ/kg and easy to store and transport. Direct combustion for combined heat and power (CHP) in sugar mills is currently the most advance and large-scale utilization method, enabling energy self-sufficiency. Excess electricity can be integrated into the grid, providing significant economic and environmental benefits [82]. A typical mill can meet over 90% of its steam and power needs via bagasse CHP, with a surplus of approximately 30–50 kWh/t of cane. Building on this, advanced energy utilization pathways are rapidly developing. Anaerobic digestion can convert bagasse into biogas, which is purified into biomethane for heating, power, or vehicle fuel. Research indicates that pretreatment can enhance methane yield by 30–50% [83]. Furthermore, thermochemical conversions, such as pyrolysis and gasification, transform bagasse into CO and H_2_ syngas, which can be further catalytically reformed into advanced liquid fuels or hydrogen [84,85]. Unlike conventional incineration, modern bagasse-to-bioenergy biorefineries deliver multi-dimensional technical advantages. Their superiority transcends simple profitability, actively driving total-resource cascading valorization, high-precision emission mitigation, and advanced product functionalization.

#### 3.2.2. High-Performance Bio-Based Materials

Bagasse-based biomaterials represent the most active area of research and offer the highest-value products. Key products include nanocellulose, bio-activated carbon, and construction and composite materials. The techno-economic performance of different sugarcane bagasse high-value utilization pathways varies greatly, and the comparative analysis results are shown in Table 3. The abundant cellulose resources in sugarcane bagasse serve as a critical prerequisite for achieving high-value biomass utilization, which necessitates the separation of lignin from cellulose. Various pretreatment strategies have been developed in current literature, including organic acid methods, biphasic system methods, deep eutectic solvent (DES) methods, and combinations of steam explosion with organic solvents. By disrupting the complex cross-linked networks among lignin, cellulose, and hemicellulose, these approaches enable the efficient fractionation of individual components [86,87,88,89,90,91,92,93,94].

##### Nanocellulose

Nanocellulose derivatives extracted from sugarcane bagasse are primarily classified into cellulose nanocrystals (CNCs) and cellulose nanofibers (CNFs) based on isolation mechanisms [98]. Cellulose nanocrystals (CNCs) are primarily produced via acid hydrolysis, where acids selectively degrade the amorphous regions of cellulose fibers, leaving behind highly crystalline, rod-like nanoparticles (NPs). Characterized by high rigidity and the ability to self-assemble into chiral nematic liquid crystals, CNCs serve as unique nanofillers for enhancing the properties of polylactic acid (PLA) [99,100,101]. When blended with polylactic acid (PLA), functionalized CNCs (e.g., P-CNCs) catalyze high-temperature dehydration and carbonization to form a stable protective char layer, significantly bolstering nanocomposite thermal stability [100,101]. Conversely, CNFs are isolated through intense physical shear forces, such as high-pressure homogenization or cavitation—yielding highly networked fibril suspensions with prominent thickening and reinforcing properties [98,102]. Notably, ultrasound-assisted extraction of CNFs from bagasse can elevate material crystallinity by 25%, positioning them as potent reinforcing agents for advanced polymer composites [103].

Distinct from top-down chemical or physical isolation, biological synthesis offers a unique bottom-up alternative to fabricate high-purity bacterial cellulose (BC) with complex nanostructuresy [104,105,106]. This metabolic process utilizes specialized aerobic strains, predominantly from the genus *Komagataeibacter*, to polymerize carbon sources like glucose or fructose at the cell surface [107,108,109]. Extracellular cellulose synthase complexes assemble these monosaccharides into β-1,4-glucan chains, which subsequently interweave into a resilient three-dimensional nanofiber network [109]. These diverse isolation and biosynthetic pathways strictly dictate the final nanocellulose morphology, crystallinity, and surface chemistry, thereby defining their performance limits in advanced composites and aerogels [98].

##### Bio-Activated Carbon

Bagasse-based activated carbon (AC) is produced by pyrolysis and activation of sugarcane bagasse, yielding a high specific surface area and well-developed pore structure. Pyrolysis involves the thermal decomposition of biomass under oxygen-limited conditions; specifically, slow pyrolysis is preferred for maximizing biochar yield [110]. Furthermore, magnetic biochar composites can be synthesized from bagasse via streamlined one-step pyrolysis method [111]. Activation is the critical stage for imparting high porosity and adsorption capacity. Chemical activation employs reagents, such as KOH or H_3_PO_4_. For instance, using KOH at 800 °C with an impregnation ratio of 1:4 can yield AC with an extraordinary specific surface area of 3554 m^2^/g, which is noticeably higher than the values reported for typical graphene-based porous carbons (3355–3523 m^2^/g) [112]. Alternatively, physical activation using steam or CO_2_ at 900 °C can produce AC with a surface area of about 489 m^2^/g [96]. These bagasse-derived materials offer a sustainable solution for high-performance adsorption and environmental remediation [113,114].

The high specific surface area and electrical conductivity of bagasse-derived activated carbon have garnered significant attention in the electrochemical field. This activated carbon features a microporous structure and high surface area, exhibiting excellent double-layer capacitance. Through modifications, such as high-energy ball milling, its specific capacitance can be increased up to 257 F/g [115]. Furthermore, after loading with CoO, the specific capacitance improved from 89.53 to 102.04 F/g [116]. Lithium-ion capacitors assembled with this material as the cathode have demonstrated an energy density of 28.4 Wh/kg and specific capacitance of 141.8 mF/g, maintaining 75% capacity retention after 100 cycles, highlighting its promising application prospects [117].

Bagasse-based activated carbon also finds extensive applications in catalysis and adsorption. It serves as an effective support for enzyme immobilization (e.g., lipase), achieving immobilization efficiencies of up to 100%, which significantly enhances enzyme reusability and stability [118]. When modified through sulfuric acid sulfonation, the resulting activated carbon possesses Lewis and Brønsted acid sites, functioning as an efficient acid catalyst for the selective dehydration of xylose into the platform chemical furfural [119]. For adsorption, powdered activated carbon can be granulated with biopolymers, such as sodium alginate and gelatin. By incorporating bagasse ash to enhance mechanical strength, these materials are transformed into granular adsorbents that are easy to deploy and regenerate. Such adsorbents have demonstrated exceptional performance in dye removal, maintaining over 90% adsorption efficiency even after seven adsorption-desorption cycles [96]. This rotational capability underscores a more robust and stable adsorption performance than that of standard commercial granular counterparts.

#### 3.2.3. High-Value Platform Chemicals

Bagasse hemicellulose and cellulose can be hydrolyzed into xylose and glucose, respectively, which serve as precursors for conversion into high-value chemicals, such as xylo-oligosaccharides (XOS), xylitol, and lactic acid. This pathway underpins the integrated sugarcane biorefinery architecture.

##### Xylo-Oligosaccharides

Xylo-oligosaccharides (XOS) are functional prebiotic oligosaccharides comprising 2–7 xylose units linked byβ-1,4-glycosidic bonds, typically extracted from the hemicellulose (xylan) fraction of lignocellulosic biomass [120,121]. Prevalent manufacturing methodologies include physical, chemical, and biological pathways, each defined by distinct trade-offs in efficiency, purity, and environmental footprint (Table 4) [122].

Physical methods utilize thermal or mechanical energy to hydrolyze xylan. Autohydrolysis or steam explosion leverages high-temperature water to cleave glycosidic bonds, yielding up to 50.35% XOS from sugarcane bagasse at 200 °C [123], while coupling hydrothermal pretreatment with GH10 endo-xylanase elevates yields to 96 mg/g [124]. Emerging intensification technologies, such as sonophotocatalysis and ultrasound-assisted extraction, radically compress processing times to selectively generate low-molecular-weight XOS (DP 2–4) [125]. Chemical methods offer highly cost-effective industrial alternatives via acid or alkali treatments. To bypass the severe sugar degradation and furfural inhibition typical of traditional inorganic acids, mild organic acid hydrolysis (e.g., citric, maleic, or acetic acid) is increasingly preferred; for instance, citric acid treatment yields 52.3% prebiotic XOS [126], whereas maleic acid coupled with enzymes achieves a 60.5% bagasse-to-XOS conversion [127]. Alternatively, alkaline extraction serves as an efficient upstream option to deconstruct biomass, solubilizing up to 86% relative xylan from bagasse using NaOH [128].

Biological methods rely on microbial xylanases for the specific, mild hydrolysis of xylan into high-purity XOS with minimal byproduct formation. For example, crude xylanase from *Bacillus subtilis* enables low-cost, targeted conversion of ammonia-pretreated bagasse [129]. Furthermore, integrated stepwise processes (e.g., combining hydrothermal, xylanase, acid, and cellulase treatments) drive total-biomass valorization. This coordinated approach achieves a 54.5% XOS yield—predominantly high-value xylobiose and xylotriose (>70%) while simultaneously maintaining a glucose enzymatic hydrolysis efficiency above 85% from the leftover cellulose matrix, successfully anchoring a zero-waste biorefinery chain [120].

##### Xylitol

Xylitol is a natural five-carbon sugar alcohol with a sweetness profile comparable to sucrose, characterized by its low caloric content and non-cariogenic properties, making it highly sought after in the food, oral care, and pharmaceutical industries. Industrially, it is primarily produced using sugarcane bagasse hemicellulose hydrolysate as a feedstock, where xylose is bioconverted into xylitol by microorganisms, particularly yeasts [130,131]. While traditional yeasts like *Saccharomyces cerevisiae* generally cannot utilize xylose efficiently, numerous non-conventional yeasts have been widely employed for xylitol production. These include *Candida tropicalis* [132,133], *Candida guilliermondii* [134,135], *Debaryomyces hansenii* [136,137], and *Pichia fermentans* [138].

Xylitol-producing yeasts exhibit a robust capacity for converting xylose while tolerating inhibitors present in hydrolysates. For instance, *Candida guilliermondii* has achieved a high yield of 0.9 g/g [134], while *Candida tropicalis*, utilizing bagasse enzymatic hydrolysate, reached a concentration of 62.98 g/L [139]. To ensure high quality, supercritical CO_2_ extraction has been employed to recover xylitol with a purity of 99.59% [140]. Technological innovations have further optimized production efficiency. Using sugarcane bagasse as an immobilization carrier has significantly improved cell reusability [135,141]. Moreover, the implementation of a complementary strain system (e.g., *C. tropicalis* paired with *B. subtilis*), where the bacteria consume byproducts and detoxify the medium, allowed the yeast to focus on conversion, resulting in a xylitol titer of 95 g/L and conversion rate of 74% [142].

##### Other Fermentation Products

However, sugarcane bagasse hydrolysate can be utilized to produce other high-value derivatives, such as lactic acid and phenylacetylcarbinol (PAC). Regarding lactic acid fermentation, the newly isolated *Pediococcus pentosaceus* strain HLV1 has successfully produced lactic acid using sugarcane field residues [43]. Furthermore, PAC, a critical pharmaceutical intermediate, can be efficiently synthesized by fermenting bagasse hydrolysate with specific yeast strains [143].

#### 3.2.4. Challenges

Although sugarcane bagasse is abundant and renewable, its recalcitrant lignocellulosic structure severely constrains efficient biorefining. Traditional pretreatments, such as acid, alkali, and steam explosion methods suffer from high energy consumption and heavy capital costs, while generating hazardous byproducts. Despite the immense market potential of nanocellulose, xylooligosaccharides, and xylitol, their production costs remain 3–5 times higher than petrochemical alternatives due to the prohibitively expensive pretreatment, enzymatic hydrolysis, and purification processes, ultimately resulting in low overall resource utilization.

### 3.3. Resource Utilization and Safety Management of Filter Mud

Filter mud is a solid organic byproduct produced during the clarification stage of sugarcane juice processing. It is rich in organic matter and essential nutrients, such as nitrogen, phosphorus, potassium, calcium, magnesium, and iron, and contains 6.70–11.01% crude wax [15]. These characteristics endow it with significant agricultural value and industrial potential. However, due to the possible presence of heavy metals, pathogens, and pesticide residues, its utilization must prioritize safety, efficiency, and high-value conversion.

#### 3.3.1. Agricultural Fertilizers and Soil Amendment

Filter mud is a significant byproduct of the sugarcane juice clarification process, primarily composed of crude fiber, ash, and essential minerals [144]. Agricultural recycling remains the most traditional and widely implemented method for its utilization. Direct application of filter mud to soil enhances organic matter content (OMC), improves soil aggregate structure, reduces acidity, and increases water and nutrient retention, thereby significantly promoting crop growth and development [145]. Compared with conventional commercial mineral fertilizers, the granular organic-inorganic fertilizer formulated with filter mud as an organic source showed no significant differences in key indicators, such as sugarcane stalk yield and total sugar content, under recommended application rates, thereby achieving equivalent substitution. Data from two consecutive crop harvests demonstrated that the filter mud-based fertilizer could stably maintain sugarcane productivity without compromising industrial processing qualities, including juice purity and fiber content [146]. Research indicates that applying filter mud increases cation exchange capacity (CEC), water-holding capacity (WHC), and microbial diversity. Studies evaluating various application ratios in clay loam soil found that filter mud effectively improves soil quality and sugarcane yields [147,148]. In Indonesia, its use as an organic fertilizer in sugarcane cultivation has been shown to boost soil fertility and overall productivity [149]. Furthermore, when applied with phosphate fertilizers, filter mud enhances phosphorus availability, optimizes rhizosphere microbial community structures, and mitigates phosphorus leaching [150].

Agronomic filter mud deployment poses critical heavy metal risks. Bioavailable metals (Cd, Pb and Zn) accumulate persistently in soil, driving selective crop uptake and shoot-ward translocation. In sandy soil, mud applications significantly elevate Zn, Cu, Pb and Cd bioaccumulation in sugar beets and carrots [151]. Major knowledge gaps persist regarding long-term migration trajectories, bioavailability regulation, and safe loading thresholds. Defining these eco-safety boundaries is imperative. To further enhance efficacy and safety, filter mud often undergoes pretreatments, such as composting, bio-carbonization, or alkaline activation. Composting filter mud with bagasse, poultry manure, or rice bran effectively eliminates pathogens and degrades harmful substances, resulting in high-quality organic fertilizers [152]. Specifically, using microbial inoculants derived from mangroves for filter mud composting significantly boosts process efficiency, producing bio-organic fertilizers that substantially increase soil organic carbon (SOC), available phosphorus, available potassium, and available nitrogen [153]. Thermochemical conversion also offers high-value options; co-pyrolyzing filter mud with distillery wastewater (vinasse) produces biochar-based fertilizers characterized by high nutrient recovery and strong water retention. These fertilizers are rich in P, K, Ca, and Mg, making them ideal for amending sandy and acidic soil [154,155]. Additionally, alkaline-activated potassium persulfate oxidation can rapidly convert filter mud into fulvic acid-like, high-efficiency fertilizers that simultaneously provide nutrients and passivate heavy metals in the soil [156].

#### 3.3.2. Preparation of High-Value Products

Sugarcane wax, extracted from filter mud or dissolved air flotation mud, is a high-value byproduct with wax content reaching up to 30% in flotation mud [157]. It is rich in bioactive lipids, primarily octacosanol, which serves as a key quality indicator. Octacosanol exerts potent cholesterol-lowering effects by modulating HMG-CoA reductase, specifically by reducing its mRNA transcription and protein expression while accelerating its degradation [158]. Beyond lipid regulation, it exhibits antioxidant, anti-inflammatory, and anti-atherosclerotic properties by inhibiting LDL oxidation and enhancing HDL functionality [159]. Recent studies using zebrafish models have shown that policosanol promotes brain cell growth and wound healing, while preliminary gastric cancer models suggest its potential to inhibit tumor cell viability and growth [160,161].

In industrial and materials science, specific additives derived from filter mud oils can be formulated into bio-greases with performance comparable to that of industrial lubricants [162]. Furthermore, filter mud ash has been used to replace cement partially; when combined with microbially induced calcium carbonate precipitation (MICP), it yields composites with high mechanical strength and low porosity [163]. Additionally, filter mud serves as a sustainable calcium source for the synthesis of carbonated hydroxyapatite. When combined with bacterial cellulose, it forms bone tissue-engineering scaffolds with excellent biocompatibility and enhanced protein adsorption [164].

#### 3.3.3. Challenges

Although filter mud is rich in organic matter and nutrients, its industrial application is hindered by severe safety and technical barriers. The presence of heavy metals and pesticide residues in filter mud poses potential ecological and food safety risks, which restricts its direct agricultural application. Furthermore, the extraction efficiency of crude wax is limited to only 5–8% due to inefficient separation technologies and low native wax content. However, the optimum moisture content and compositional instability of filter mud complicate large-scale drying and processing, thereby constraining its overall economic value.

## 4. Conclusions and Future Perspectives

Based on the ‘Sugarcane Processing Tree’ framework, this article comprehensively summarizes the high-value utilization technologies and industrial status of sugarcane juice, bagasse, and filter mud. This study mainly concluded the sugarcane juice industrialization is shifting from legacy sugar and ethanol toward high-value upgrading. Highly profitable functional sugars anchor this transition, led by D-allulose ($100,000/ton; 15–20 folds sucrose prices) and fructooligosaccharides. Concurrently, abundant polyphenols and antioxidants are valorized into natural preservatives and low-GI sweeteners. Sugarcane bagasse dominates biomass yields, maximizing commercial hemicellulose-to-xylitol/XOS conversion. However, biorefining faces severe economic bottlenecks. Pretreatment compromises 40–50% of total costs. Additionally, high-value nanocellulose costs 3–5 times more than petrochemical alternatives, severely restricting industrial scaling. Filter mud is nutrient-rich but bottlenecked by heavy metal risks and low crude wax recovery (5–8%). Direct agronomic utilization remains heavily restricted. Consequently, converting filter mud into biochar or organic fertilizer represents the more useful and safe valorization pathway.

Based on a comprehensive synthesis and critical analysis, the future sugarcane research must resolve critical biological and processing bottlenecks. For juice processing, engineering high-sugar/ethanol-tolerant strains and implementing enzyme engineering will stabilize fermentation and slash costs. Establishing rigorous juice quality standards is essential to mitigate seasonal variations and contamination risks. For bagasse biorefining, developing green, low-cost pretreatments, such as deep eutectic solvents must pair with optimizing xylo-oligosaccharides and xylitol co-production. Concurrently, advancing lignin valorization will eliminate cost barriers and maximize resource efficiency. Finally, for filter mud utilization, developing heavy metal stabilization and detoxification techniques will guarantee agronomic safety. Optimizing separation processes must overcome low crude wax extraction efficiency, ultimately establishing standardized frameworks to drive industrial-scale scaling.

## Figures and Tables

**Table 1:** Comparison of typical deep-processing products and technologies of sugarcane juice.

Category	Specific Product	Core Processing Technology	Technical Maturity (TRL)	Main Application Fields	Source
Basic sugar products	White granulated sugar	Clarification, concentration, crystallization, centrifugal separation, drying	TRL 9	Beverage, food processing, catering, daily consumption, bulk commodity trading	[16]
	Refined sugar	Multi-step impurity removal and non-sugar component purification	TRL 9	High-end food, infant formula, medical preparations	[17]
	Rock candy	Redissolving and recrystallization of white granulated sugar	TRL 9	Seasoning, traditional tonics, medicinal raw materials	[18]
	Soft white sugar	White granulated sugar compounded with invert sugar syrup	TRL 9	Pastry making, direct household consumption in North China	[19]
	Non-centrifugal brown sugar	Primary clarification, evaporation and crystallization without deep decolorization	TRL 9	Traditional desserts, flavoring, health-care food	[20,21,22]
	Pharmaceutical sucrose	High-purity refining, deep impurity removal and sterile treatment	TRL 9	Pharmaceutical excipients, stabilizer for bioactive components	[23,24]
Functional sugars	D-allulose	Enzymatic epimerization via Izumoring strategy, microbial biosynthesis	TRL 7–8	Low-sugar food, beverage, healthy sugar substitute	[25,26,27]
	Fructo-oligosaccharides (FOS)	Enzymatic synthesis, enzyme molecular modification, dual-enzyme synergistic catalysis	TRL 7–8	Prebiotics, infant formula, feed additives, health products	[28,29,30]
	High-fructose corn syrup (HFCS)	Glucose isomerization	TRL 9	Beverages, baking, dairy	[31,32]

Note: TRL -Technology Readiness Level: TRL 9 represents mature industrialized production; TRL 7–8 represents laboratory mature technology with ongoing industrial demonstration and scale-up.

**Table 2:** Comparison of fermentation products from sugarcane juice.

Product	Strain	Yield/Concentration	Fermentation Duration	Main Industrial Application	Source
Ethanol	*Saccharomyces cerevisiae*	8–12% (*v*/*v*)	24–72 h	Biofuel, beverage industry	[40,41,42]
Lactic acid	*Lactobacillus plantarum*	50–80 g/L	48–96 h	PLA synthesis, food additive	[43,44]
Succinic acid	*Issatchenkia orientalis*	60–100 g/L	72–120 h	Polymer, pharmaceutical	[45,46,47]
Sugarcane wine	*Saccharomyces cerevisiae*	5–12% (*v*/*v*)	7–21 d	Alcoholic beverage	[48,49,50]
Protopanaxadio	Engineered *S. cerevisiae*	15.88 g/L	120 h	Pharmaceutical precursor	[51]

**Table 3:** Techno-economic comparison of high-value products from sugarcane bagasse.

Product	Major Function	Market Value (USD/t)	Key Advantages	Source
Nanocellulose	Pretreatment + energy	52,000–552,500	High strength, biodegradable	[95]
Activated carbon	Activation reagents	276–1242	High adsorption capacity	[96]
XOS	Enzyme hydrolysis	2182–4500	Prebiotic, high market demand	[97]
Xylitol	Fermentation + purification	2509	Low-calorie sweetener	[97]

**Table 4:** Comparison of common methods for xylo-oligosaccharide production from sugarcane bagasse.

Application	Principle	Advantages	Disadvantages	Yield	Source
Physical	Steam explosion/autohydrolysis	Fast, easy scaling	High energy consumption	50.35%	[123,124,125]
Chemical	Organic acid hydrolysis	Mild, less by-products	Corrosive, high cost	52.3–60.5%	[126,127,128]
Biological	Xylanase hydrolysis	Mild, high purity	Slow, enzyme cost	54.5%	[120]

## Data Availability

No data was used for the research described in the article.
